# Results of Delayed Primary Suture and of Secondary Suture

**Published:** 1918-03

**Authors:** H. A. Ballance


### RESULTS OF DELAYED PRIMARY SUTURE AND OF SECONDARY SUTURE

By Colonel H. A. BALLANCE, R. A. M. C.

I have been asked to bring to your notice the results of delayed primary and secondary suture in the hospitals of Etaples and Camiers, but I do not know whether the account I have to give is one which should be presented at all to a meeting like this, because, as regards the great majority of the wounds that have been closed in those hospitals which I visit, there has not been any pathological research into the bacteriology of the wounds at the time of their closure. It is the practice of the great majority of the surgeons in my district, when closing a wound, to dispense with a bacteriological investigation of it, and I will give you results.

We have listened to Captain Morrison's report with interest, and he will, I am sure, be the first to recognize that in the special surgical research wards to which he is attached he is under certain great advantages; and one of these consists in the fact that in these wards there is a permanent medical and nursing staff and orderly personnel which are not available for other duties. Owing to the great shortage of medical officers in the English service hospitals, a permanent staff cannot be guaranteed in them, and men are liable to be transferred from time to time to other wards and other hospitals as their services may be required. Investigation under these circumstances is very difficult, but I have been astonished at the amount of patient investigation which some surgeons at Etaples and Camiers have been able to do in spite of difficulties.

I am not going to occupy your time very long, because it seems to me that at these meetings the active workers should do by far the greater part of the talking.

A great deal of delayed primary and secondary suture is done at Etaples and Camiers, and I have tried to make an estimate of the number of wounds sutured last year. Probably quite 1,000 were sutured during the year in the hospitals in that district, in which we have over 20,000 beds. I am afraid the investigation of hospital records is a depressing exercise. I have been through very many hundreds and I have had to cast aside a great many as not giving information of any value. As to records of secondary suture of wounds, I have often come across a record of this kind : Date. Patient admitted to base hospital. Amputation of the thigh at C.C.S. Wound in a fair condition. Then comes another entry, say a fortnight later : Wound sutured ; then another date : Evacuated to England.

I have not included in my account cases recorded in this way, although it may well be that such a case would not have been evacuated unless going on well; but I have as a rule included only those records in which were described the condition of the wound, the method of treatment, the number of days observed after the date of primary or secondary suture as the case may be, and the condition when evacuated.

Many men seem to think that secondary suture of a wound is such an ordinary part of the day's surgical work that they really do not trouble to make any special note of the procedure, and that is, I think, why the notes are often so fragmentary.

The majority of the wounds in the Etaples district are not examined bacteriologically before undergoing secondary suture. The appearance of a wound, of its margin, and of the discharge, is noted; also the character of the surrounding soft parts and the general condition of the patient, and, if all these are satisfactory, the majority of the surgeons will consider themselves justified in closing such a wound without seeking the aid of a bacteriological count. It is astonishing to me what freely suppurating wounds are sometimes closed successfully, even when complicated by a fresh cut section of bone left in the depths of the closed wound. There are a great many surgeons, who, if they find they are able to secure a large proportion of successes in secondary suture of wounds, without the aid of a microbial count, will go on trusting to their naked eye diagnosis to the end of the chapter.

There are, however, records from a few surgeons who have controlled their secondary suture work bacteriologically. These are records, as a rule, of the Carrel-Dakin treatment. I do not want to burden you with figures, but a few may not come amiss.

One great difficulty I have in estimating the value of any particular chemical reagent used in the treatment of a wound is the most annoying way some men have of mixing their antiseptics. I have a record of 100 wounds treated by one surgeon — quite a good one — who used the Carrel-Dakin method and in a great majority of the cases did not suture the wound until it reached the Carrel suture standard; but when it did reach that, he would, at the operation of closure, smear the wound all over with bismuth and iodoform paste and then suture it. In 50 o/o of these cases he obtained union by first intention; 25 o/o were complete failures. It really looked in that series of cases as though B. I. P. P. had had a particularly malign influence on the healing of the wounds. 65 o/o were flesh wounds and 35 o/o bone. Many were of considerable severity.

Of cases of delayed primary suture there were 21; of secondary suture there were 79; and in this series of 100 cases, although the flesh wounds were much more numerous than the bone, 11 o/o of the flesh wounds were failures, but 55 o/o of the bone wounds broke down. When you are talking to the man who believes in B. I. P. P., and he admits to you that a case has failed, it must be remembered that it is really " some " failure, because the whole wound breaks down and the healing process has to start anew.

The influence of pathological control was well shown in this series of 100 cases. There were 19 o/o of failures in cases from which smears were taken and counts made, but there were 41 o/o of failures where counts were not made,

A much better series of 63 sutures of wounds was given me by two very careful workers using the Carrel-Dakin method. They had 3 o/o failures and 84 o/o healing by first intention.

Most men, when asked what proportion of successful secondary sutures they have when using B. I. P. P. to the wounds, will say, " Yes, certainly ", in answer to the question, " Can you suture successfully 9 out of 10 ? " They certainly believe they get 90 o/o of successes, but that is not always borne out on examining the records.

One series of 56 cases, all of which were flesh wounds except 5, had 9 failures — that is, 16 o/o. These 56 cases were almost equally divided between delayed primary and secondary suture. There were 3 failures in delayed primary suture and 6 in secondary.

When you talk about a successful result or a good result after the use of B. I. P. P., it does not necessarily mean union by first intention. I do not think the advocates of B. I. P. P. would wish to suggest that this is the usual method of union. At the end of a few days, as many know, there is often some considerable discharge between the stitches holding the edges of the wound together. That discharge may go on for days, with some amount of surrounding inflammation and a rise of temperature. In the favorable cases that discharge will disappear in a week to ten days, often rather suddenly at the end, and the wound will then heal very rapidly.

I have also analysed the records of 118 cases of suture, using B. I. P. P., by one very good surgeon in the Etaples district, with this result : — Out of 118 cases there were 10 failures, just under 9 o/o which, it seems to me, is quite a good result. He is a very careful surgeon and would not say his cases were successes unless they were. Four bone cases were failures and six flesh wounds. Of the 118 cases 36 were fractures, 82 were wounds of the soft parts. I have seen in the practice of this surgeon fractures of the humerus, of the bones of the forearm and of the leg, sutured by this method — both delayed primary or secondary suture — and I have been surprised how many wounds healed uneventfully. Of this 118, 65 were delayed primary suture with 4 failures, that is to say, suture carried out within the first five days; 53 were secondary suture with 6 failures.

I am afraid I am rather wearying you with all these figures. Another series of 79 cases had 23 bone wounds and 56 wounds of the soft parts. There were 74 good results and 5 failures. 43 of them were delayed primary suture with 2 failures, 1 bone and 1 flesh wound; the rest, secondary with 3 failures, all flesh wounds.

I really do not think that we have yet any reliable scientific evidence to show that this much recommended B. I. P. P., which is used so freely at Etaples, enables a surgeon to sew up a wound successfully which, without it, he would have been unable to close. If this substance is any real good at all, this ought to be demonstrable ; but although there are many hundreds of wounds being sutured in this way in the Etaples hospitals each year, yet we really do not know what is going on in the wounds; and I think that one case carefully treated and carefully observed in the way in which Captain Morrison is accustomed to treat and observe his cases is worth at least as much as any ten of those I have brought to your notice this afternoon.

I only regret that I have appeared before you as a mere news agent rather than a worker.

